# Addressing medical student burnout through informal peer-assisted learning: a correlational analysis

**DOI:** 10.1186/s12909-024-05419-w

**Published:** 2024-04-26

**Authors:** Paola Campillo, Frances Ramírez de Arellano, Isabel C. Gómez, Natalia Jiménez, Joan Boada-Grau, Legier V. Rojas

**Affiliations:** 1 School of Medicine, Universidad Central del Caribe, Bayamón, Puerto Rico, USA; 2 Cellular-Molecular Biology Dept, University of Puerto Rico (RP), San Juan, Puerto Rico, USA; 3 Interdisciplinary Sciences Dept, University of Puerto Rico (RP), San Juan, Puerto Rico, USA; 4 Education Sciences and Psychology Dept, Universitat Rovira I Virgili, Av. Catalunya, 35, 43002 Tarragona, Spain; 5 Physiology Dept. School of Medicine, Universidad Central del Caribe, 100 Av. Laurel, Bayamón, Puerto Rico, 00956 USA

**Keywords:** Medical students, Academic burnout, Peer assisted learning, Informal peer assisted learning, School burnout inventory

## Abstract

**Background:**

Despite the recognized advantages of Peer-Assisted Learning (PAL) in academic settings, there is a notable absence of research analyzing its effects on students' Academic Burnout. This study aims to cover this gap by assessing the underlying effectiveness of Informal Peer-Assisted Learning (IPAL) as a cooperative learning method, focusing on its potential to mitigate academic burnout among medical students.

**Methods:**

In 2022, a cross-sectional study was conducted at the School of Medicine, Universidad Central del Caribe, in Puerto Rico. The research team gathered data from 151 participants, 49.19% of 307 total student body. This cohort included 76 female students, 71 male students, and 4 individuals saying other. The School Burnout Inventory questionnaire (SBI-9) was employed to assess Academic Burnout, along with an added query about self-reported IPAL. The SBI-9 underwent validation processes to ascertain its reliability and validity, incorporating the Exploratory Factor Analysis and Confirmatory Factor Analysis. Following this, the investigators conducted an analysis to determine the correlation between academic burnout levels and involvement in IPAL.

**Results:**

The validation process of the questionnaire affirmed its alignment with an eight-item inventory, encapsulating two principal factors that elucidate academic burnout. The first factor pertains to exhaustion, while the second encompasses the combined subscales of cynicism and inadequacy.

The questionnaire shows high reliability (Cronbach's alpha = 0.829) and good fit indices (Comparative Fit Index = 0.934; Tucker-Lewis Index = 0.902; Standardized Root Mean Squared Residual = 0.0495; Root Mean Squared Error of Approximation = 0.09791; *p*-value < 0.001). The factors proven in the selected model were used to evaluate the correlation between Academic Burnout and IPAL. Students engaged in IPAL showed significantly lower academic burnout prevalence compared to those who never participated in such practices, with a mean academic burnout score of 44.75% (SD 18.50) for IPAL engaged students versus 54.89% (SD 23.71) for those who never engaged in such practices (*p*-value < 0.013). Furthermore, within the group engaged in IPAL, students displayed lower levels of cynicism/inadequacy 41.98% (SD 23.41) compared to exhaustion 52.25% (SD 22.42) with a *p*-value < 0.001.

**Conclusions:**

The results of this study underscore a notable issue of academic burnout among medical students within the surveyed cohort. The investigation reveals a significant correlation between Academic Burnout and IPAL, suggesting that incorporating IPAL strategies may be beneficial in addressing burnout in medical education settings. However, further research is needed to explore potential causal mechanisms.

**Supplementary Information:**

The online version contains supplementary material available at 10.1186/s12909-024-05419-w.

## Background

Burnout, characterized by overwhelming mental and physical exhaustion, presents a critical concern within the medical student community. This phenomenon is strongly associated with reduced feelings of achievement and depersonalization, potentially leading to adverse student outcomes, such as poor academic performance, compromised mental health, increased dropout rates, and even suicidal ideation [1] [2]. A correlation between burnout and academic performance has been demonstrated, with burnout emerging as a negative predictor of academic achievement across various measures such as exams, grades, and GPA, reaffirming the importance of addressing burnout to safeguard students' academic success and overall health [3] [4].

The nine-item School Burnout Inventory (SBI-9) questionnaire supplies a standardized tool for assessing academic burnout (ABO), encompassing three key sub-scales: exhaustion (EX), cynicism (CY), and inadequacy (IN) [5]. These metrics, along with others, have been instrumental in shaping our understanding of burnout as a psychological syndrome [6] and have contributed to the International Classification of Diseases-11 definition, characterizing burnout as an occupational phenomenon resulting from chronic workplace stress that has not been effectively managed [7].

The prevalence of ABO among medical students has been on the rise, evidenced by a 6% increase in burnout levels in the United States from 2008 to 2014 [8], with estimates suggesting that half of all medical student's worldwide experience ABO even before entering residency [9]. Preliminary research conducted at the Universidad Central de Caribe (UCC) also showed elevated levels of ABO among its medical students [10].

Despite various support systems implemented by medical schools [11] [12] [13] effective strategies to mitigate ABO are still lacking. Recognizing that students and healthcare professionals experiencing burnout are more susceptible to unprofessional behavior, it is imperative to promote and supply effective support mechanisms to mitigate ABO [14] [15].

Recent research has shown that the learning environment significantly influences ABO rates among medical students, with lower learning environment scores correlating with higher burnout rates [16]. In this context, Peer-Assisted Learning (PAL), has been identified as effective strategies for enhancing student wellness [17] [18] [19] [20] [21]. PAL encompasses a spectrum of peer-to-peer educational activities, including near-peer assisted learning, where more experienced students guide their less-experienced counterpart [21]. This approach has been shown to foster essential skills such as problem-solving, critical thinking, and effective communication [11] [22] [23].

Informal PAL (IPAL), unlike its formal counterparts, develops organically through social networks and study groups among students, fostering a unique environment for collaborative learning and knowledge exchange without direct faculty or institutional oversight [24]. Although lacking a formal structure, IPAL offers opportunities for knowledge exchange and collaborative learning, contributing to students’ learning outcomes and overall academic success [25]. Additionally, it enhances students' self-efficacy, coping skills, and social support networks, all essential for academic success [26]. Research shows that peer learning improves students' comprehension of the subject matter and boosts their confidence in their roles [27].

While PAL is recognized for its various advantages in academic settings, there remains a gap in literature concerning its impact on students’ ABO. This lack of research highlights a crucial area of investigation, particularly in the high-pressure environment of medical education. Building upon this framework, our investigation is directed towards two primary objectives. Initially, we aim to estimate the ABO within our cohort of medical students and secondly, we seek to evaluate and elucidate the relationship between ABO and IPAL among these medical students. Guided by these aims, our research is driven by two primary questions: (1) Can the SBI-9 be considered a valid and reliable tool for assessing ABO in our context? and (2) What is the correlation between ABO and IPAL among medical students? By addressing these questions, our study aims to contribute to the broader understanding of strategies for mitigating burnout in medical education and offer evidence-based recommendations for promoting IPAL in medical education. Partial results from this study were presented at the December 2022 conference of the Medical Association of Puerto Rico [28].

## Methods

### Survey: measurement tools

We conducted a cross-sectional study using the nine-item School Burnout Inventory (SBI-9), administered online to assess Academic Burnout (ABO) among medical students [5]. Participation was voluntary, with students self-reporting their gender, age range, and academic standing. The Institutional Review Board (IRB) of the UCC approved the method and corresponding protocols (054–2022-25–06-IRB).

The SBI-9 was provided in both its original English form [5] and a Spanish-adapted version [29] to meet the bilingual needs of our university context (refer to Supplementary Material 1). We followed established standards for translating and adapting assessment instruments [30].

The SBI-9 questionnaire, which is freely available for research purposes, was chosen to assess ABO due to its strong psychometric properties and its comprehensive approach in university settings. The SBI-9 is specifically structured into three subscales: Exhaustion (EX) with four items, Cynicism (CY) with three items, and Inadequacy (IN) with two items. These sub-scales enable a nuanced examination of the several factors of ABO, assisting in the identification and reduction of potential confounding factors that contribute to student burnout.

### Rating scale

Participants rated each SBI-9 item on a Likert scale from 1 (complete disagreement), 2 (disagree), 3 (neutral), 4 (agree) to 5 (complete agreement). In this instance, the purpose was to restrict the capacity to capture subtle nuances in students' opinions, opting instead for a concise representation on the five-scale value.

### Measurement of informal peer-assisted learning

We evaluated IPAL engagement through a single item, asking students about the frequency of explaining concepts to peers during informal study sessions. In this study, we sought to assess participant’s engagement in IPAL to understand informal collaborative learning behaviors among medical students. In order to measure IPAL, a single question (in Spanish and English) regarding the frequency with which they explain concepts to their peers during their study sessions was included, expressed as “Aunque estudie solo(a) generalmente explico los conceptos a mis compañeros”; alternatively, "Although I study alone, I usually explain concepts to my colleagues” (Before the questionnaire was submitted, the students agreed that the word colleagues referred to their classmates). Responses were categorized as 'never' (NE) = 0, 'occasionally' (O) = 3, and 'frequently' (F) = 5. In interpreting the results, responses for the behavior of IPAL were grouped into two categories: those who indicated they 'never' (NE) engaged in the behavior and those who responded 'occasionally' or 'frequently' (O/F). This grouping strategy was implemented after consideration of the distribution of responses and nuances in students’ opinions.

### Study sample

In January 2022, we conducted a cross-sectional study involving a study sample of 151 participants, representing 49.19% of the medical student population (*n* = 307) at the UCC in Puerto Rico. This sample size provides a study confidence level exceeding 90% with a 5% margin of error. Among these participants, 76 identified as female, 71 as male, and 4 did not specify their gender. The inclusion criteria encompassed medical students in their 1st to 4th year, aged 21 years or older. Additional demographic information alongside their corresponding ABO levels and parameters are detailed in Supplementary Material 2.

### ABO Calculations

The overall ABO calculation was carried out using the eight-item version of the SBI (SBI-8) [31], with high ABO defined as averages above 50%. For graphical analyses, data were aggregated and analyzed from the entire sample population, merging English and Spanish responses, and Likert scale values of each responder were converted into percentages, which were then averaged and statistically processed.

### Statistical analysis

The process of establishing the factors influencing ABO involved several key steps.

We initiated our statistical approach with a Principal Component Analysis (PCA) to discern the main components contributing to ABO. Following PCA, we conducted Exploratory Factor Analysis (EFA) and validated our findings through Confirmatory Factor Analysis (CFA), referencing Gaussian Graphical Models [32] for additional insight (see Supplementary Material 3).

Cronbach's alpha was used to assess the internal consistency reliability of the scales, providing a measure of the extent to which all the items in the scale are correlated to each other. For validating the SBI in our medical student cohort, we adhered to Hu and Bentler's (1999) [33].

The EFA, performed using Jamovi for Windows, followed procedures modeled after Coşkun et al. (2023) [34]. We initially assessed data suitability for factor analysis by examining the correlation matrix and applying Bartlett’s Test of Sphericity alongside the Kaiser–Meyer–Olkin Measure of Sampling Adequacy (KMO MSA).

Our preliminary assessment evaluated the correlation matrix. Conducting factor analysis does not make sense if there is no correlation between items over 0.30 [35]. Correlation values (Spearman’s Rho) among items exceeded the threshold (except for item-EX3, that was excluded from the analysis), indicating adequacy for the EFA. In our case, we allow correlation greater than 0.2, although not very high, since it indicates that there is some relationship between the variables and, given the nature of the data, the inclusion of these variables in a factor analysis is justified by the Bartlett’s Test of Sphericity (χ2 = 430, *p* < 0.001) and a satisfactory KMO MSA value of 0.815, confirming the dataset’s appropriateness for factor analysis. Both results showed that the data has no inadequacy to carry out factor analysis [35].

In determining the optimal number of factors, we employed three strategies: (a) Eigenvalue cut-off rule, (b) the “elbow” joint in the scree plot, and (c) fixed number. Direct Oblimin, an oblique rotation technique, was deemed suitable for our study given the norm of factor intercorrelation in social sciences studies [36]. We accepted 0.40 level as a factor loading threshold to consider that a factor is stable [37].

EFA identified significant factor loadings, with values for Factor-1 (EX) ranging from 0.30 (minimum acceptable) to 0.78, and for Factor-2 (CYIN) from 0.53 to 0.84. Subsequent PCA supported these findings, indicating component loadings from 0.43 to 0.88 for component 1, and 0.71 to 0.85 for component 2.

A two-factor model emerged from the EFA: Factor 1 encompassing EX and Factor-2 combining CY and IN (CYIN). CFA evaluated this model, with goodness-of-fit indices suggesting a well-fit model: CMIN/df 2.45, CFI (Comparative Fit Index) 0.93, TLI (Tucker‐Lewis Index) 0.90, RMSEA (Root Mean Square Error of Approximation) 0.098 (0.06–0.13), SRMR (Standardized Root Mean Square Residual) 0.05. Standardized regression weights varied between 0.42 and 0.72, affirming the model’s stability and relevance, evidenced by a Cronbach’s alpha coefficient of 0.828.

To visually present our findings regarding ABO, we used GraphPad Prism v.9. Additionally, we performed more analyses, including Pearson coefficient and Ordinary One-way ANOVA. For showing the Exploratory and Confirmatory Factor Analysis, as well as Multiple Correlation Comparisons and Path Model Mediation, we used Jamovi v2.3 with R subroutines (The Jamovi Project, 2022, https://www.jamovi.org).

## Results

### Student burnout inventory validation

The internal consistency of the SBI-9 was confirmed through a correlation matrix and Cronbach’s alpha, which revealed a high reliability coefficient of 0.913. PCA was conducted to identify the underlying structure within the data, choosing the most suitable model based on eigenvalues greater than 1 and a factor loading threshold of 0.4, using Oblimin rotation to facilitate interpretation. We explored the data with EFA to identify the underlying structure. This analysis revealed two loaded components: one composed of EX items (excluding EX3) and the other combining CY and IN items into a singular CYIN component. Both the significance of Bartlett's Test of sphericity (χ2 = 430, *p* < 0.001) and the KMO MSA (0.825, range 0.788–0.855) confirmed the data’s suitability for factor analysis.

The exclusion of item-EX3 due to minimal correlation within the EX-subscale of ABO (Tables 1 and 2), and the fusion of CY with IN creating the Fc2, was further confirmed by the Gaussian Graphical Model (GGM) [32] (refer to Supplementary Material 3). This led to the adoption of the Puerto Rican version of the SBI, now referred to as SBI-8, with item-EX3 removed for subsequent analyses. In all instances, our results align with models (refer to Table 3), excluding item- EX3 who demonstrated elevated uniqueness (0.94; CI 0.91–0.97).

**Table 1:** Correlation Matrix of the SBI-9 and the overall Reliability of the SBI-8 and per Items (excep EX3)

**Correlation Matrix**																	
	**EX1**		**CY1**		**IN1**		**EX2**		**CY2**		**CY3**		**EX3**		**IN2**		**EX4**
**EX1**	—																
**CY1**	0.34233	***	—														
**IN1**	0.37281	***	0.57814	***	—												
**EX2**	0.31982	***	0.25650	**	0.24792	**	—										
**CY2**	0.25428	**	0.65773	***	0.56691	***	0.32925	***	—								
**CY3**	0.30156	***	0.64923	***	0.43755	***	0.36810	***	0.69321	***	—						
**EX3**	0.10740		-0.01058		0.01020		0.21068	**	0.01009		0.11084		—				
**IN2**	0.25808	**	0.32807	***	0.48843	***	0.15284		0.50232	***	0.36703	***	0.15983	*	—		
**EX4**	0.20292	*	0.26473	**	0.31372	***	0.38855	***	0.32686	***	0.34476	***	0.11997		0.25170	**	—

Items Correlation Matrix and Reliability statistics. Data derived from Jamovi v2.2.2. Asterisks in the correlation matrix highlight statistically significant values as detailed in the table's footnote. EX3 subfactor has larger no significant correlation among sub-parameters. Two scales of global reliability are presented Cronbach’ α. The sub-parameters item’s reliability is presented in Cronbach’ α values only

^*^ *p* < .05, ** *p* < .01, *** *p* < .001

**Table 2:** Correlation Matrix of the SBI-9 and the overall Reliability of the SBI-8 and per Items (excep EX3)

Scale Reliability Statistics (SBI-8) no EX3				
	mean	SD	Cronbach's α	
Scale	2.8572	0.78948	0.829	
Item Reliability Statistics				
	**mean**	**SD**	**Item-rest correlation**	**Cronbach's α**
EX1	3.7285	1.0324	0.417	0.825
CY1	2.3046	1.2165	0.655	0.796
IN1	3.1589	1.2388	0.638	0.798
EX2	2.8212	1.2654	0.419	0.827
CY2	2.3642	1.2935	0.725	0.785
CY3	2.3113	1.3226	0.678	0.791
IN2	3.2848	1.3681	0.485	0.819
EX4	2.8411	1.3520	0.429	0.826

Items Correlation Matrix and Reliability statistics. Data derived from Jamovi v2.2.2. Asterisks in the correlation matrix highlight statistically significant values as detailed in the table's footnote. EX3 subfactor has larger no significant correlation among sub-parameters. Two scales of global reliability are presented Cronbach’ α. The sub-parameters item’s reliability is presented in Cronbach’ α values only

**Table 3:** Bivariate Correlation Models and the statistics under the Confirmatory Factor Analysis

MODEL						RMSEA 90% CI						
ESTIMATED	Models (SBI-9)	CFI	TLI	SRMR	RMSEA	Lower	Upper	AIC	BIC	χ2	df	p
M1e	1F(CYINEX)	0.976	0.967	2460	0.478	0.000	0.0845	5822	5904	36.4	27	0.107
M2e	2F-a (CYIN-EX)	1.000	1.020	2331	0.000	0.000	0.0000	5807	5892	19.3	26	0.822
M3e	3F (CY-IN-EX)	1.000	1.050	2524	0.000	0.000	0.0000	5802	5893	10.2	24	0.994
M4e	2F-b (EXIN-CY)	1.000	1.010	2956	0.000	0.000	0.0554	5811	5896	23.2	26	0.624
M5e	2F-c (CY-EX)	1.000	1.020	0.085	0.000	0.000	0.0580	4917	4983	9.3	13	0.750
**FINALS**	**Models (SBI-8)**	**CFI**	**TLI**	**SRMR**	**RMSEA**	**Lower**	**Upper**	**AIC**	**BIC**	**χ2**	**df**	**p**
M1	1F(CYINEX)	0.904	0.866	0.0589	0.1150	0.0820	0.149	3637	3709	60	20	< 0.001
M2	2F-a (CYIN-EX)	0.934	0.902	0.0495	0.0979	0.0625	0.134	3626	3701	47	19	< 0.001
M3	3F (CY-IN-EX)	0.951	0.920	0.0416	0.0888	0.0497	0.128	3620	3702	37	17	< 0.003
M4	2F-b (EXIN-CY)	0.932	0.899	0.0505	0.0995	0.0643	0.135	3627	3702	47	19	< 0.001
M5	2F-c (CY-EX)	1.000	1.030	0.0898	0.0000	0.0000	0.044	4419	4476	4	8	= 0.891

Statistical values of the Confirmatory Factor Analysis (CFA) and model fix. M1 one factor model in which all subscales (CY, EX, and IN) are grouped into one factor. Three (3) two models’ factors (M2, M4 and M5). In M2, CY and IN subscale are grouped into one factor, EX maintain as second factor. In M4, EX and IN are groped in one factor, CY maintains as second factor and in M5, where CY and EX represent the factors. The M3 represents the three factors model in which CY, EX and IN are factors. 1F represents one factor model, 2F represents two factors model and 3F represents three factors model. 2F three different models (a, b and c)

*χ2* = chi-square, *df* = Degrees of freedom, *CFI* = Comparative fit index, *TLI* = Tucker–Lewis index, *RMSEA* = Root mean square error of approximation, *p* = *p*-value

CFA further validated these findings, supporting the configuration of the two-factor model as most representative of our data (Table 3). This model, detailed in Table 3, effectively captures the dimensions of ABO within our medical student cohort. Operating under this premise, we evaluated five models of the SBI-8, as delineated in Table 3, to identify the model that most accurately aligns with our observed results.

The five models presented various configurations, as displayed in Table 3, with Model M2 from the SBI-8 appearing as the most suitable. In Model M2, CY and IN items were combined as one factor (Fc2), while EX items formed another (Fc1). The analysis showed that ABO, as measured by the SBI-8 in model M2, proved the most robust statistical consistency. The CFA and reliability analysis yielded a Cronbach’s α of 0.927, signifying excellent internal consistency. The high KMO (measure of sample adequacy) value for Model M2 (> 0.82) confirmed excellent sample adequacy for all eight items. Model M2’s χ2, TLI, CFI, RMSEA, and SMRS, with a *p*-value < 0.001, showed a good fit to the data (refer to Table 3).

### Academic burnout in medical students

**Fig. 1:**
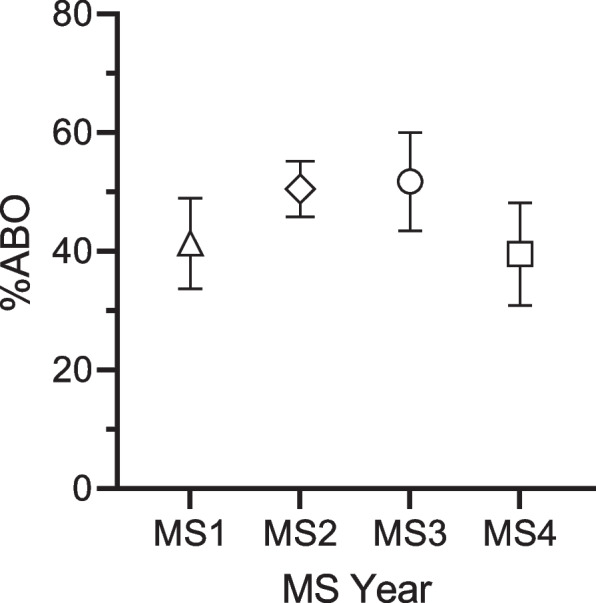
The Academic Burnout per academic year of medical students. The ABO values for both MS1 and MS4 were lower compared to MS2 and MS3. Furthermore, the proportion of students with ABO scores above 50% in each year was as follows: 10/38 (26.32%), 29/56 (51.79%), 14/27 (51.85%), and 10/30 (33.33%) for MS1, MS2, MS3 and MS4, respectively. The ABO scores across four different medical student (MS) years, specifically from the 1st year (MS1) to the 4th year (MS4), along with their corresponding 95% confidence intervals (CI). The calculated ABO percentages, represented as mean percentage (standard deviation) and sample size, for each year were as follows: MS1, 41.34 (23.19) 38; MS2, 50.50 (17.44) 56; MS3, 51.75 (20.98) 27; and MS4, 39.54 (23.06) 30

The data collected from our survey, analyzed under the two-factor Model M2 derived from the SBI-8 (as depicted in Table 3), allows for precise categorization of ABO percentages among participants by academic year. The analysis revealed no statistically significant variation in ABO values across academic years, from first year (MS1) to fourth year (MS4) (refer to Fig. 1).

From Fig. 1 the mean ABO percentages, standard deviation (in parenthesis), and the number of respondents and their percentage (in parenthesis) for each academic year respectively were as follows: MS1, 41.34 (SD 23.19) for 38 (25%); MS2, 50.50 (SD 17.44) for 56 (37%); MS3, 51.75 (SD 20.98) for 27 (18%) and MS4 39.54 (SD 23.06) for 30 (20%), from the sample population *N* = 151.

The percentage of students with ABO values above 50% in each year is as follows: MS1, 26.32%, (10 out of 38 respondents); MS2, 51.79% (29 out of 56 respondents); MS3, 51.85% (14 out of 27 respondents); and for MS4, 33.33% (10 out of 30 respondents).

Our gender-based analysis showed no significant differences in ABO levels: males reported an average ABO of 44.76% (SD 19.16, *n* = 71) and females 48.68% (SD 23.45, *n* = 76). Similarly, language preferences—Spanish (47.31%, SD 21.82, *n* = 111) or English (47.36%, SD 20.94, *n* = 40)—did not significantly impact ABO scores. Additional demographic details are available in Supplementary Material 2.

Four students who did not disclose their gender, showing an average ABO of 60.42% (SD 11.42, *n* = 4), were excluded from the gender-specific analysis due to the small sample size.

### Analysis of factors contributing to academic burnout in medical students

**Fig. 2:**
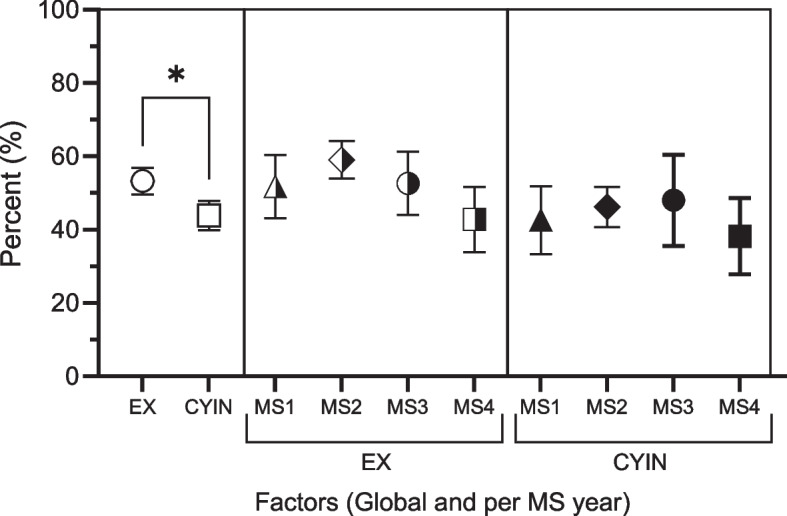
Factors contributing to Academic Burnout (ABO). Contribution of Factors Fc1 (EX) and Fc2 (CYIN) globally (left part) and per academic year right part. The figure shows average percentages, and 95% confidence intervals (CI) for the two factors. On the far left and with clear symbols are the overall percentages standard deviation and number of students. The percentage, standard deviation and number of values were obtained after excluding the EX3 item based on the final M2 model: Fc1 (EX) represented by circle 53.26 (22.40) *N* = 151, Fc2 (CYIN), represented by square 43.88 (24.68) *N* = 151. The global percentage between these two factors is statistically significant. On the right are represented with mean symbols half-full the percentages of the Fc1, EX for each year of study: triangle MS1, 51.75 (26.16) *N* = 38; rhombus MS2, 59.081(9.22) *N* = 56; circle MS3, 52.65 (19.48) *N* = 22; and square MS4, 42.82 (23.44) *N* = 29. Following in that order are the percentages of the Fc2, CYIN factor with fully filled symbols for each year of study: MS1, 42.60 (28.28) *N* = 38; triangle, MS2, 46.21 (20.61) *N* = 56; rhombus, MS3, 48.01 (28.15) *N* = 22; circle and MS4 square 38.24 (26.83) *N* = 28

Reliability analysis for the SBI-8, assessed with Cronbach’s α-coefficient, showed high internal consistency (α = 0.829). Importantly, the analysis indicated CYIN-factor (Fc2) consistently showed lower values compared to the global EX-factor (Fc1), represented as an empty circle and square, respectively (Fig. 2). This difference was statistically significant (*p*-value < 0.01), as illustrated in Fig. 2 (left). However, when comparing EX and CYIN percentages across medical school years, no distinct difference emerged between these two factors (see Fig. 2, right).

### Diminished academic burnout in medical students engaged in informal peer assisted learning

**Fig. 3:**
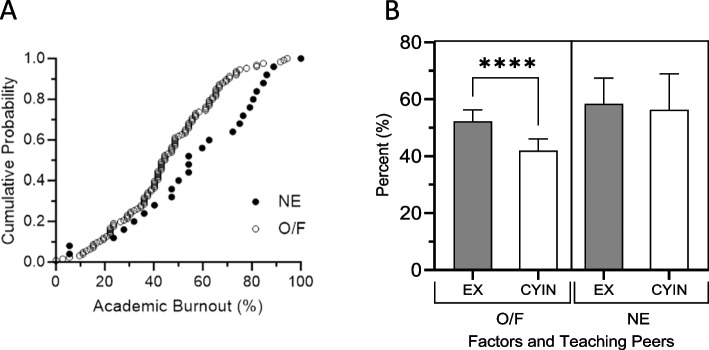
**A** The academic burnout percent value of each medical student in the population’s offering peer-teaching (O/F) and those never do that (NE). The academic burnout percent value of each medical student (MS) in the population’s offering peer-learning (O/F) and those never do that (NE). The figure shows cumulative probability of the percentages of academic burnout (ABO) within the medical student population, specifically those who indicated that they never taught their peers—NE (Fill circles) and those who reported doing so frequently or occasionally—O/F do informal peer learning (clear circles). Results presented excluding the EX3 item based on the findings of the CFA (model M2). The O/F student group is shifted to the left, indicating a lower average ABO value. The mean percentage values, standard deviations (SD), and sample sizes for the O/F population were 44.75 (18.50) *N* = 126, while for the NE population they were 54.89 (23.71) *N* = 25. The O/F population had a statistically significant lower proportion of academic burnout compared to the NE population (*p* < 0.0133). **B** Factors Fc1 and Fc2 (EX and CYIN) involved in academic burnout and the relationship with students who taught their peers (O/F) and those of students who did not informally tutor their peers (NE). Factors (EX and CYIN) involved in academic burnout. In the left part, the figure shows the relationship of students who do informal peer learning (IPAL) to their peers (O/F) and those who did not do so (NE). When analyzing the students who do IPAL, the percentage of Fc2 is statistically lower *p* < 0.001 compared to the Fc1: 41.98 (23.41) vs 52.25 (22.42) *N* = 126. On the other hand, in students who do not take IPAL, there is no significant difference in the percentages of Fc2: 56.33 (30.65) vs Fc1: 58.33 (22.05) *N* = 25. Values represent, the mean percentage values, standard deviations (SD), and sample sizes

As depicted in Figs. 3A, our results indicate that medical students engaged in IPAL experience lower levels of ABO compared to their peers who reported no engagement in tutoring their peers. Specifically, students reporting occasional or frequent engagement in IPAL (O/F) displayed an ABO score of 44.75% (SD 18.50) for 126 (83% of the respondents) students, lower than the 54.89% (SD 23.71) observed for the 25 (17% of the respondents) students who never engaged in IPAL (NE). This difference was statistically significant, with a *p*-value of 0.0133.

Further examination of the two-factor Model M2, as presented in Fig. 3B, highlights that the reduction in ABO among IPAL-participating students is particularly pronounced in the factor CYIN (Fc2), which was significantly lower than the EX-factor (Fc1) (*p*-value < 0.001).

Figure 3B delineates the detailed breakdown of these factors, comparing the percentages for each between students who engaged in IPAL O/F versus those who did not (NE). The results show that Fc1: EX for the O/F group was 52.25% (SD 22.42) for 126 (83%) of the respondents, lower than the NE group’s 58.33 (SD 22.05) for 25 (17%) of the respondents. Similarly, Fc2: CYIN for the O/F group was 41.98% (SD 23.41) for 126 (83%) of the respondents, less than the NE group’s 56.33% (SD 30.65) for 25 (17%) of the respondents.

## Discussion

Our findings validate the use of the School Burnout Inventory (SBI) for our sample. The validation process confirmed the SBI-8's alignment with an eight-item inventory (SBI-8), with two principal factors of ABO: EX and a combined measure of CY and IN (CYIN). Notably, this two-factor Model M2 (employing the SBI-8) emerged as the most proper (Table 3), consistent with findings from other studies using the SBI-9 and SBI-8 [38] [39]. The validated model underscores the interrelated nature of CY and IN, suggesting common underlying issues, such as a lack of support or resources at school, or a mismatch between students’ skills and academic demands. This model has implications for interventions aimed at reducing burnout, as addressing one factor may help alleviate the other. For example, interventions that aim to improve students' skills and resources, or to better match students with their academic jobs, could potentially alleviate both CY and feelings of IN. Noticeably, this two-factor model supplies a simplified and potentially more actionable framework for understanding and addressing ABO among medical students. However, further research is needed to fully understand ABO and find the most effective interventions for alleviating it.

The prevalence of ABO in our medical school mirrors levels reported in medical schools across the United States [1] [40]. Despite our school’s abundance of support resources and emphasis on the availability of help, the persistent ABO underscores a notable issue of ABO among medical students within the surveyed cohort. This pattern is not unique to our institution but reflects a broader challenge faced by many educational institutions [11] [41] [42].

Our study introduces a unique perspective by delving into the role of IPAL on the experiences of ABO among medical students, offering valuable insights into this critical issue. The pivotal finding is the significant (*p*-value < 0.013) decrease in ABO levels among medical students who engage in IPAL, compared to those who do not (Fig. 3A), from 44.75% (SD 18.50) for IPAL engaged students versus 54.89% (SD 23.71) for those who never engaged in such practices. Moreover, our analysis reveals that medical students engaged in IPAL show a significant reduction (*p* < 0.001) in the combined levels of CY and IN (O/F-CYIN) compared to EX (O/F-EX), as illustrated in Fig. 3B. This translates into a significant (*p* < 0.001) reduction in ABO among students participating in IPAL (O/F—IPAL) compared to those do not participate at all (NE—IPAL). This finding suggests the potential of IPAL as mitigating factor against ABO in our academic environment.

Furthermore, our findings suggest that the factors Fc2 (CYIN) and Fc1 (EX) are linked to increased ABO levels in students who reported never (NE) taking part in IPAL (Fig. 3B). While the specific mechanisms behind this association were not the focus of our initial study, the observed correlation prompts a deeper investigation. The fact that students with lower ABO levels may be more predisposed to engage in IPAL raises questions about the direction of this relationship. Given the significance of this finding, further detailed studies are called for to understand the causality behind these dynamics.

Preliminary analyses, as outlined in Supplementary Material 4, show that IPAL directly reduces ABO, particularly by diminishing the levels of the CYIN (or Fc2) aspect rather than through a mediating effect on overall ABO. This effect contrasts with a common assumption about mediating factors: instead of indirectly affecting overall ABO through different paths, IPAL directly targets and reduces the specific elements of CY and IN. The statistical significance of IPAL’s direct impact on CYIN suggests that its effect is not due to random chance. Therefore, we recommend that interventions aiming to reduce ABO should prioritize IPAL, focusing specifically on lowering CY and IN (Fc2). Further examination reveals that while IPAL significantly affects the CYIN component of ABO, its influence on the EX-component (Fc1) is minimal or non-existent (refer to Supplementary Material 4), which suggests IPAL's benefits may be more psychological and social than physical or emotional. This distinction is critical because it adds insights into potential strategies to mitigate ABO levels in medical students. Therefore, further research is needed to develop a comprehensive understanding of ABO and how IPAL can play a role in its alleviation. [1] [9]. While many studies have shed light on factors that mitigate ABO, none has specifically discussed the impact of peer learning on ABO.

Our findings demonstrate that students doing IPAL either occasionally or frequently (O/F) exhibit significantly lower levels of CYIN when compared to their levels of EX. This distinction underlines the potential of IPAL as a targeted strategy to address specific components of ABO. However, earlier studies have highlighted the dynamic nature of peer learning, that a student's enthusiasm for and engagement in peer learning can vary over time [19] [43] [44], which could impact the effectiveness of IPAL. Through regular IPAL assessments, it could be possible to proactively show and address these fluctuations, implementing the right interventions to sustain their benefits. By fostering a supportive community that encourages collaboration, IPAL has the potential to significantly reduce ABO. This, in turn, enhances learning efficiency and helps students develop effective coping strategies, thus addressing the multifaceted nature of ABO by offering psychological, social, and academic support [17–21], [45–47].

## Limitations

Our study has several limitations. First, due to its cross-sectional design, it lacks a control group, limiting our capability to make temporal comparisons concerning ABO rates and other aspects of medical students’ well-being throughout their careers. Future studies should consider longitudinal designs to enable more effective comparisons over time.

Second, our study encountered limited medical student participation, with a 49.19% (151 out of 307 medical students), which introduces the potential for response rate bias. This bias may affect the results if, for example, students experiencing higher levels of distress were either less likely or more likely to participate, due to the subject matter's pertinence. However, such patterns were not evident in our analysis.

Third, our research was conducted at a single medical school, restricting the generalizability of our findings to the broader medical student population in Puerto Rico.

Lastly, the nature of our questionnaire limited our ability to collect comprehensive psychological and personal data from the students, thus narrowing the study’s overall depth. Future studies should consider exploring a broader array of factors, such as studying conditions, to provide a more holistic understanding of the ABO experiences among medical students.

## Conclusions

Our research presents compelling evidence of a widespread ABO issue among medical students in our study population, with observed levels alarmingly aligning with trends seen in medical schools throughout the United States. This issue underscores an urgent need for immediate and targeted intervention strategies to mitigate these ABO levels.

In addressing our first research question, our findings confirm that the SBI, particularly the SBI-8, serves as a valid and reliable instrument for assessing ABO in the context of our study. This validation offers a foundation for accurately measuring ABO levels among medical students.

Turning to our second research question, the data reveals a significant correlation between ABO and IPAL. Our data indicates that students engaged in IPAL, whether occasionally or frequently, exhibit notably lower levels of cynicism and inadequacy, two critical dimensions of ABO. This finding not only reaffirms the value of IPAL as an academic practice but also positions it as a viable method for reducing elements of ABO among medical students. Given this correlation, we advocate for the promotion of IPAL within medical curricula as a proactive approach to reduce ABO.

### Supplementary Information

**Supplementary Material 1.**

## Data Availability

The datasets used and/or analyzed during the current study are available from the corresponding author upon reasonable request.

## Abbreviations

ABO: Academic Burnout
CFA: Confirmatory Factor Analysis
CFA: Confirmatory Factor Analysis
CY: Cynicism
CYIN: Cynicism/Inadequacy
EFA: Exploratory Factor Analysis
EX: Exhaustion
Fc1, Fc2: Factor 1, Factor 2
GGM: Gaussian Graphical Model
IN: Inadequacy
IPAL: Informal Peer Assisted Learning
MS1, MS2, MS3, MS4: Medical students’ year 1, 2, 3 and 4, respectively
NE: Never
O/F: Occasionally / Frequently
PCA: Principal Component Analysis
SBI-8: School Burnout Inventory -8 items
SBI-9: School Burnout Inventory -9 items
