# Supplementary material for: Addressing medical student burnout through informal peer-assisted learning: a correlational analysis
Source: BMC Med Educ. 2024 Apr 26;24:460. doi: 10.1186/s12909-024-05419-w (PMC11055289; doi:10.1186/s12909-024-05419-w)
Supplement: Supplementary file 1 — Supplementary Material 1. [file 12909_2024_5419_MOESM1_ESM.docx]

**Supplementary Material**

**Supplementary Material 1.**


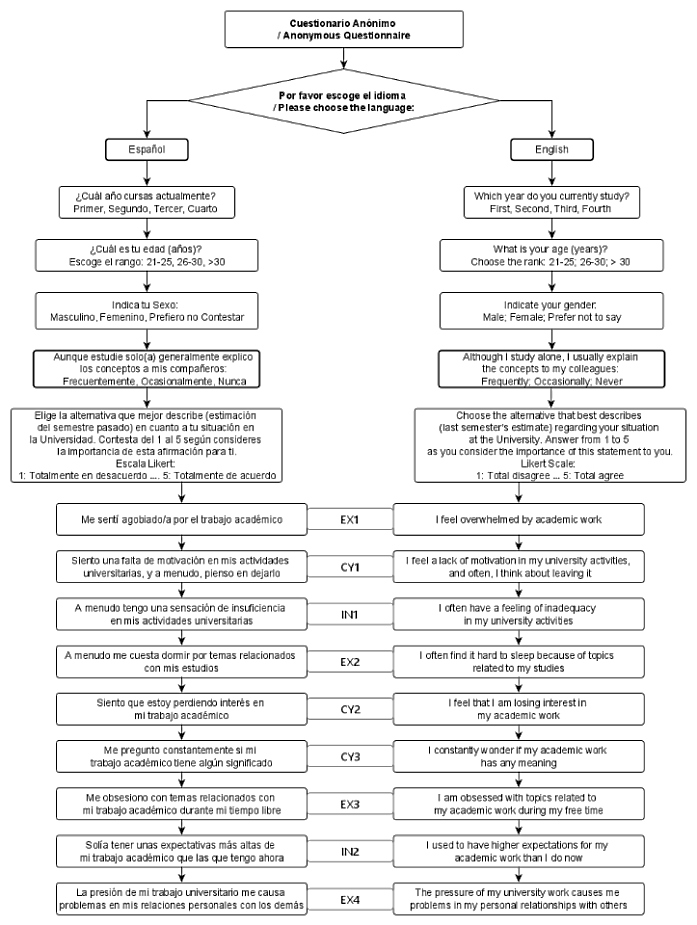


**Supplementary Material 1.**

Questionnaire sequence and the School Burnout Inventory-9 (SBI-9). The diagram illustrates the structured sequence of queries in the questionnaire. Initially, participants choose their preferred language. This is followed by a series of demographic questions including their academic year, age group, and gender. Subsequently, the questionnaire addresses a key topic on Informal Peer Assisted Learning, before delving into the core section comprising the nine questions of the School Burnout Inventory (SBI-9).

**Supplementary Material 2.**

| LANGUAGE |  | EX-SP | CY-SP | IN-SP | ABO-SP | EX-EN | CY-EN | IN-EN | ABO-EN |
| --- | --- | --- | --- | --- | --- | --- | --- | --- | --- |
|  | N | 111 | 111 | 111 | 111 | 40 | 40 | 40 | 40 |
|  | Mean (%) | 53.68 | 33.18 | 55.07 | 47.31 | 52.08 | 33.13 | 56.88 | 47.36 |
|  | SD (%) | 22.41 | 28.63 | 27.51 | 21.82 | 22.63 | 27.25 | 30.08 | 20.94 |
|  |  |  |  |  |  |  |  |  |  |
| GENDER |  | FEM | MALE | FEM-SP | MALE-SP | FEM-EN | MALE-EN | PNR-SP | PNR-EN |
|  | N | 76 | 71 | 59 | 50 | 17 | 21 | 2 | 2 |
|  | Mean (%) | 48.684 | 44.758 | 50.448 | 43.112 | 42.565 | 48.677 | __ | __ |
|  | SD (%) | 23.453 | 19.156 | 23.548 | 19.38 | 22.736 | 18.474 | __ | __ |
|  |  |  |  |  |  |  |  |  |  |
| AGE (range) |  | ≤25 | ≥26 | ≤25 SP | ≥26 SP | ≤25 EN | 26≥ EN |  |  |
|  | N | 98 | 48 | 82 | 29 | 16 | 19 |  |  |
|  | Mean (%) | 49.901 | 42.448 | 49.628 | 40.757 | 51.301 | 45.029 |  |  |
|  | SD (%) | 20.121 | 23.86 | 20.374 | 24.67 | 19.342 | 22.981 |  |  |

**Supplementary Material 2.** The summary of demographic data is segmented by language (upper section), gender (middle section), and age (lower section). The percentage means of ABO and their parameters EX, CY and IN presented by language (upper part), gender (middle part), and age (lower part). In every category, rows present the number of values, the mean percentage, and the standard deviation, respectively. Regarding language, the percentages for the parameters of exhaustion (EX), cynicism (CY), and inadequacy (IN) are specified for both Spanish (SP) and English (EN). For gender and age, the overall academic burnout percentage (%ABO) is calculated using the SBI-8 in which EX3 is excluded. In the gender category, SP and EN denote Spanish and English, respectively, while PNR represents students who preferred not to specify their gender. In the age category, all students were categorized into two age groups: ≤25 and ≥26. Across all categories, no significant statistical differences were observed when analyzed using the Ordinary One-way ANOVA for Multiple Comparisons. The Mean (%) indicates the average values for each parameter (EX, CY, IN) in Spanish (SP) or English (EN), or the % of ABO as applicable. The number of values (N), Mean (%), and Standard Deviations (SD) are detailed for each category.

**Supplementary Material 3.**


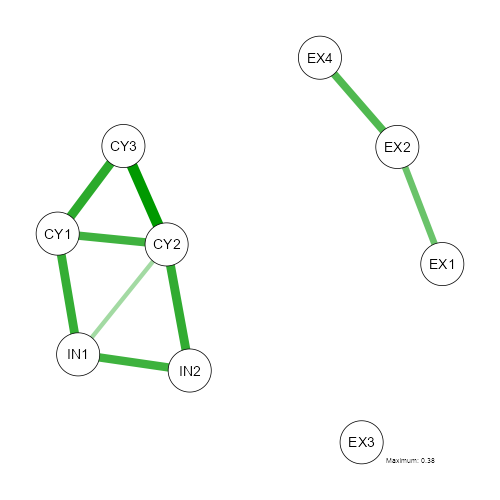


**Supplementary Material 3.** Analysis using the Gaussian Graphical Model showing the factors determine the evaluation of ABO. The Gaussian Graphical Model is a novel exploratory analysis tool that presents a roadmap to explore relationships between items and variables in environmental psychology research **[32]**. In this network model, nodes symbolize entities. Thick edges denote the intensity of interaction, reflecting the robustness of the connection. Edges link elements within the same component. For instance, in exhaustion (EX), elements EX1, 2, and 4 are interconnected, whereas EX3 remains unconnected or does not pertain to any component. The elements of cynicism and inadequacy are interconnected by edges, signifying their belongingness to the same entity. The figure presents the Gaussian Graphical Model obtained from the sample data separated by parameters defining ABO. This figure provides a detailed visualization of the interconnected elements influencing the factors that shape the ABO, as determined by the SBI-9. Factor 1 (Fc1) is constituted by items 1, 2, and 4, which represent aspects of exhaustion (EX). In contrast, Factor 2 (Fc2) emerges from the merger of items 1, 2, and 3 of cynicism (CY), in conjunction with items 1 and 2, representing inadequacy (IN). This confluence of items, representing cynicism and inadequacy, is collectively called CYIN. Exhaustion item 3 (EX3) is outside the interconnectivity of factors.

**Supplementary Material 4.**

|  |  |  |  | **95% C.I. (a)** | |  |  |  |
| --- | --- | --- | --- | --- | --- | --- | --- | --- |
| **Type** | **Effect** | **Estimate** | **SE** | **Lower** | **Upper** | **β** | **z** | **p** |
| Indirect | EX ⇒ IPAL ⇒ ABO | -2.46e−4 | 8.53E-04 | -0.00192 | 0.00143 | -2.56e−4 | -0.288 | 0.773 |
|  | CYIN ⇒ IPAL ⇒ ABO | 0.00163 | 0.00132 | -9.64e−4 | 0.00422 | 0.00192 | 1.231 | 0.218 |
| Component | EX ⇒ IPAL | 0.03563 | 0.12136 | -0.20224 | 0.2735 | 0.02712 | 0.294 | 0.769 |
|  | IPAL ⇒ ABO | -0.00689 | 0.00464 | -0.01599 | 0.00221 | -0.00943 | -1.485 | 0.138 |
|  | **CYIN ⇒ IPAL** | **-0.23604** | **0.10729** | **-0.44633** | **-0.02575** | **-0.20323** | **-2.2** | **0.028** |
| Direct | EX ⇒ ABO | 0.33108 | 0.00693 | 0.31750 | 0.34465 | 0.34456 | 47.805 | < .001 |
|  | CYIN ⇒ ABO | 0.65975 | 0.00622 | 0.64756 | 0.67193 | 0.77668 | 106.102 | < .001 |
| Total | EX ⇒ ABO | 0.33083 | 0.00700 | 0.31712 | 0.34455 | 0.34431 | 47.281 | < .001 |
|  | CYIN ⇒ ABO | 0.66137 | 0.00619 | 0.64925 | 0.67350 | 0.77860 | 106.919 | < .001 |
| Note. Confidence intervals computed with method: Standard (Delta method). Betas are completely standardized effect sizes. | | | | | | | |  |

**Supplementary Material 4**

Evaluation of IPAL's mediation in inhibiting the development of ABO: The implications of this effect of factor 2 -Fc2 (CYIN)- on the IPAL. The table shows the lack of a mediating role played by Informal Peer-Assisted Learning (IPAL) in the context of Academic Burnout (ABO). Notwithstanding, an inverse relationship is evident between cynicism/inadequacy (CYIN) and IPAL. This is quantified by a correlation coefficient (r) of -0.23604, did a beta coefficient (β) of -0.203, and further substantiated by a statistically significant p-value of 0.028. These findings underscore that while IPAL significantly mitigates CYIN, it does not act as an intermediary in reducing ABO. Furthermore, the influence of CYIN on ABO is both direct and statistically substantial, as indicated by a p-value of less than 0.001. This preliminary result of this assessment of mediation of the component CYIN over IPAL suggests the effects are not by chance and "IPAL" should be considered in interventions or analyses aimed at influencing "ABO". A more detailed analysis including several models of mediation over the factors’ components is in course.
